# Early Postoperative Complications of Gastrointestinal Surgery and Its Associated Factors in Yemeni Patients Treated in a Teaching Hospital: A Retrospective Monocentric Study

**DOI:** 10.7759/cureus.25215

**Published:** 2022-05-22

**Authors:** Menawar Dajenah, Faisal Ahmed, Anessa Thabet, Khaled Ghaleb, Hossein-Ali Nikbakht

**Affiliations:** 1 Department of General Surgery, Ibb University of Medical Sciences, Ibb, YEM; 2 Department of Urology, Al-Thora General Hospital, Ibb University of Medical Sciences, Ibb, YEM; 3 Department of Gynecology, Ibb University of Medical Sciences, Ibb, YEM; 4 Department of Internal Medicine, Ibb University of Medical Sciences, Ibb, YEM; 5 Department of Biostatics and Epidemiology, Social Determinants of Health Research Center, Babol University of Medical Sciences, Babol, IRN

**Keywords:** small bowel resection, risk factors, complications, early postoperative, abdominal surgery

## Abstract

Background

Postoperative complications (POCs) are significant concerns to surgeons because of their possible fatality or long-term disabilities. This study aimed to investigate the early POCs of gastrointestinal surgery and its associated factors in Yemeni patients treated in a teaching hospital in Sana'a University referral hospital.

Method

A retrospective cross-sectional study from June 2016 to June 2020 was conducted at Al-Kuwait Teaching Hospital, Sana'a University, Yemen. The patients' characteristics, causative factors, primary treatment, and POCs were recorded from their medical profiles. Univariate analysis was utilized to identify the risk factors associated with gastrointestinal POCs within 30 postoperative days.

Results

The 30-postoperative day mortality was 3.6%, and major POCs occurred in 22 (20%) patients. There is no statistically significant relationship between POCs and age, sex, smoking, khat chewing, comorbidities (diabetes mellitus, anemia, jaundice, heart disease), emergency cases, drain insertion, and operative time (p ˃ 0.05). There was a significant relationship between POCs and preoperative poor nutritional status, high American Society of Anesthesiologists (ASA) grade, need for blood transfusion, major abdominal surgeries, iatrogenic injury, small bowel resection, reoperation, and history of the previous laparotomy (p ≤ 0.05).

Conclusion

There is a significant relationship between preoperative poor nutritional status, high ASA, need for blood transfusion, major abdominal surgeries, reoperation, small bowel resection, iatrogenic injury, previous laparotomy, and POCs across different gastrointestinal procedures. These factors should be assessed when auditing surgical outcomes.

## Introduction

Globally, the number of surgical procedures has increased each year [1]. For example, in 2006, more than 14 million surgeries were conducted in the United States alone [2]. Postoperative complications (POCs) have detrimental effects on many vital aspects of patients' health [3]. About 7%-15% of patients with abdominal surgeries were anticipated to have POCs, with a 0.79%-5.7% expected mortality rate [4].

The Clavien-Dindo classification characterizes a POC as any deviation from the ordinary postoperative course, ranging from non-life-threatening complications with no lasting disability to fatal consequences [5,6]. Individually, POCs could significantly influence the patient, potentially declining both quality of life and physical capacity [7]. POCs impose a significant financial burden on society in the form of extra healthcare expenses when a patient requires intensive care unit treatment, reoperation, or readmission [8].

Several studies have been conducted to highlight the depth of POCs within specific surgical subspecialties in the developed world. However, in developing countries, more studies need to evaluate the POCs in gastrointestinal surgeries and study their trend over time [8]. This study investigated the risk factors of early POCs after gastrointestinal surgery in Yemeni patients treated in Al-Kuwait's Teaching Referral Hospital of Sana'a University.

## Materials and methods

Study design

A retrospective cross-sectional study was carried out between June 2016 and June 2020 in our teaching referral hospital (Al-Kuwait Hospital, Sana'a University, Sana’a, Yemen), including gastrointestinal surgery patients. During this period, 110 patients were enrolled in our study. The exclusion criteria included patients with non-bowel-related surgery such as simple elective cholecystectomy, hernia repairs, patients on an anticoagulant, pregnant cases, renal failure cases, immunosuppressive cases, and complications occurring after postoperative 30 days and in case of admission to another hospital. Ethical clearance was obtained from the colleges' research review and ethical committee.

Data collection

Patients' demographic characteristics such as age, gender, nutritional status, comorbidities (diabetes mellitus, anemia, jaundice, heart disease), American Society of Anesthesiologists (ASA) score, previous abdominal surgery, type of surgery (emergency or elective), diagnosis, and operative findings such as surgery size (medium, major, supermajor), iatrogenic injuries, and need for blood transfusion were gathered and compared with gastrointestinal POCs occurred within 30 postoperative days [9].

Age was categorized into three categories (10-30, 31-50, and 51-80 years). Nutritional status was categorized into three types (overweight, normal weight, and malnourished). Comorbidities were included (diabetes mellitus, anemia, jaundice, and heart disease). Iatrogenic injuries are defined as any damage to an abdominal organ or vessel. The ASA score was defined as low scores (1 and 2) and high scores (3 and 4). The size of the surgical procedure was classified as follows: (a) medium-sized operations, which include appendectomy through gridiron incision, (b) major-sized operations, which include simple laparotomies for complicated appendectomies, perforated duodenal ulcers, simple gastrojejunostomy, colostomy procedures, small bowel resection, and biliary surgery without entero-biliary anastomosis, and (c) supermajor surgery, which includes biliary pancreatic surgery that involves entero-biliary anastomosis, gastrectomy cases, and colorectal cancer surgery [10,11].

Main outcome measures

Postoperative complications were considered for every patient who needed hospitalization, reoperation, or admission to the intensive care unit and occurred within 30 postoperative days after gastrointestinal surgery. We included gastrointestinal-specific outcome variables and focused on the most meaningful complications of colorectal procedures, including surgical site infection (SSI), paralytic ileus, peritonitis, intestinal obstruction, anastomotic leakage, fistulas, and wound dehiscence.

SSI has been defined according to Manilich et al. and applied to infection of the superficial or deep tissues of the surgical incision [9]. Paralytic ileus was defined as postoperative uncomplicated ileus that resolves within two to three days after surgery, and paralytic or adynamic ileus lasts for three days. Peritonitis was defined as intraoperative evidence of peritoneal surface inflammation and contaminants or infectious peritoneal fluid in all quadrants as a complication of surgical interventions due to intestinal perforation, anastomosis leakage, or perforated gastric ulcer. Every case of localized peritonitis was ruled out. Intestinal obstruction was defined as abdominal distension or pain within 30 days of surgery. The presence of dilated bowel loops with multiple fluid levels in the plain abdominal x-ray or computed tomography (CT) scan confirmed the diagnosis. Anastomotic leaks were defined as "the leakage of luminal contents from a surgical join," and a CT scan confirmed the diagnosis. Fistulas were defined as an abnormal opening or passage between two organs or between an organ and the body’s surface, and a CT scan confirmed the diagnosis. Wound dehiscence was defined as a partial or total separation of previously approximated wound edges due to a failure of proper wound healing. Postoperative mortality was not analyzed because of the small number of cases [1]. Non-colorectal complications or adverse side effects, such as pneumonia, deep vein thrombosis, acute renal failure, and urinary tract infection, were not included in the comparison analysis.

Statistical analysis

The mean and standard deviation were used for quantitative variables, and frequency and percentage were used for qualitative variables. The chi-square test was used to determine the relationship between nominal and categorical variables regarding the number of patients developed with early POCs. A p-value of 0.05 or less was considered statistically significant. All statistical analyzes were done using STATA software, version 13 (StataCorp LLC, College Station, TX).

## Results

Patients' characteristics and presentation

A total of 110 patients were included in this study, in which 68 (61.8%) patients were males and 42 (38.2%) patients were females. The mean age was 42.49 ± 17.39 years (14-75 years). The patients' characteristics and presentation are summarized in Table 1.

**Table 1:** Patient demographic characteristics

Variables	N (%)
Gender	
Male	68 (61.8%)
Female	42 (38.2%)
Age (year)	42.49 ± 17.39
Age group (year)	
10-30	39 (35.5%)
31-50	36 (32.7%)
51-80	35 (31.8%)
Khat chewing	62 (56.4%)
Smoking	46 (41.8%)
Comorbidities	
Jaundice	14 (12.7%)
Diabetes	3 (2.7%)
Bronchial asthma	1 (0.9%)
Hypertension	5 (4.5%)
Heart failure	1 (0.9%)
Liver hepatitis	1 (0.9%)
Preoperative health status	
Anemia	14 (12.7%)
Shock	2 (1.8%)
Blood transfusion	13 (11.8%)
Nutritional status	
Overweight	1 (0.9%)
Normal weight	71 (64.5%)
Malnourished	38 (34.5%)
Previous laparotomy	14 (12.7%)
Type of surgery	
Emergency	57 (51.8%)
Elective	53 (48.2%)
Malignant conditions	34 (30.9%)
Postoperative complications	
Single	5 (4.5%)
Multiple	17 (15.5%)

The primary diagnosis was a gastrointestinal obstruction in 27 (24.6%) patients (intestinal and gastric obstruction), followed by appendicitis in 21 (19.1%) patients, followed by gastric cancer in 15 (13.6%) patients. Table 2 summarizes the primary diagnosis.

**Table 2:** Patients’ primary clinical diagnosis

Variables	N (%)
Gastrointestinal obstruction	27 (24.6%)
Appendicitis	21 (19.1%)
Gastric cancer	15 (13.6%)
Perforated duodenal ulcer	10 (9.1%)
Colon cancer	8 (7.3%)
Common bile duct obstruction	11 (10.0%)
Pancreatic cancer	7 (6.4%)
Strangulated inguinal hernia	4 (3.6%)
Colostomy closure	2 (1.8%)
Others	5 (4.5%)

Emergency was found in 57 (51.8%) patients, and elective surgical conditions were found in 53 (48.2%) patients. Malignant conditions were seen in 34 (30.9%) patients, and 14 (12.7%) patients have a previous laparotomy. The most common surgical procedure was gastrojejunostomy with or without gastrectomy in 25 (22.7%) patients, followed by appendectomy either through gridiron incision or through laparotomy in 21 (19.1%) patients, then small bowel resection in 19 (17.3%) patients and colectomy in 14 (12.7%) patients. The types of operations were summarized in Table 3. Major surgical procedures account for more than half of the patients - 60 (54.5%) patients, followed by supermajor surgery in 40 (36.4%) patients, and medium-sized operations in 10 (9.1%) patients.

**Table 3:** Types of operative procedures

Operative procedures	N (%)
Gastrojejunostomy	25 (22.7%)
Appendectomy	21 (19.1%)
Small bowel resection	19 (17.3%)
Colectomy	14 (12.7%)
Choledochoduodenostomy	10 (9.1%)
Laparotomy with appendectomy	10 (9.1%)
Duodenal ulcer repair	10 (9.1%)
Triple bypass	7 (6.4%)
Colostomy	4 (3.6%)

POCs occurred in 22 (20%) patients, and multiple complications occurred more frequently than single complications (15.5% vs 4.5%). Most of the POCs were SSIs in 14 (12.7%) patients, followed by fistula in six (5.5%) patients. The POCs are summarized in Table 4.

**Table 4:** Postoperative complications of 22 (20%) patients *The complications occurred in 22 (20%) patients, and most of the patients had multiple complications (15.5% vs 4.5%).

Variables^*^	N (%)
Surgical site infection	14 (12.7%)
Fistula	6 (5.5%)
Anastomosis leak	5 (4.5%)
Iatrogenic injury	5 (4.5%)
Paralytic ileus	5 (4.5%)
General peritonitis	3 (2.7%)
Wound dehiscence	3 (2.7%)
Intestinal obstruction	2 (1.8%)

Relations between early postoperative complications and other factors

Statistically, there is no relation between POCs and age, sex, smoking, khat chewing, comorbidities such as diabetes mellitus, anemia, jaundice, heart disease, drain insertion, emergency cases, and operative time (p ˃ 0.05). There was a significant relationship between POCs and poor nutritional status, high ASA grade, need for blood transfusion, major abdominal surgeries, reoperation, iatrogenic injury, small bowel resection, and history of the previous laparotomy (p ≤ 0.05) (Table 5).

**Table 5:** Relationship between postoperative complications and patients’ characteristics and procedure details *P-values < 0.05 were considered significant. ASA: American Society of Anesthesiologists; CVD: Cardiovascular disease.

Variables		Total (110)	Complications, n (%)		P-value*
			No, n = 88 (80%)	Yes, n = 22 (20%)	
Age group (Years)	10-30	39 (35.4)	29 (74)	10 (26)	0.446
	31-50	36 (32.7)	31 (86)	5 (14)	
	51-80	35 (31.9)	28 (80)	7 (20)	
Sex	Male	68 (61.8)	55 (80.8)	13(19.2)	0.768
	Female	42 (38.2)	33 (78.5)	9 (21.5)	
Qat chewing	Yes	62 (56.3)	51 (82.2)	11 (17.8)	0.501
	No	48 (43.7)	37 (77.1)	11 (22.9)	
Smoking	Smoker	46 (41.8)	36 (78.2)	10 (21.8)	0.699
	Non-smoker	64 (58.2)	52 (81.2)	12 (18.8)	
Jaundice	Yes	14 (12.7)	11 (78.6)	3 (21.4)	0.886
	No	96 (87.3)	77 (80.2)	19 (19.8)	
Anemia	Yes	14 (12.7)	9 (64.2)	5 (35.8)	0.116
	No	96 (87.3)	79 (82.2)	17 (17.8)	
Diabetic	Yes	3 (2.7)	3 (100)	0 (00.0)	0.508
	No	107 (97.3)	85 (79.4)	22 (20.6)	
CVD disease	Non	104 (94.5)	84 (80.8)	20 (19.2)	0.105
	H T N	5 (4.6)	3 (60)	2 (40)	
	Heart failure	1 (0.9)	0 (00.0)	1 (100)	
ASA grade	ASA1-2	89 (80.9)	74 (83.1)	15 (16.9)	0.089
	ASA3-4	21 (19.1)	14 (66.7)	7 (33.3)	
Nutritional status	Normal	71 (64.6)	61 (86)	10 (14)	0.036
	Malnourished	39 (35.4)	27 (69.2)	12 (30.8)	
Blood transfusion	Yes	13 (12)	6 (46.1)	7 (53.9)	0.001
	No	97 (88)	82 (84.5)	15 (15.5)	
Operation time	<2 hours	22 (20)	20 (91)	2 (9)	0.153
	>2 hours	88 (80)	68 (77.2)	20 (22.8)	
Drain	Yes	96 (87.2)	75 (78.1)	21 (21.9)	0.198
	No	14 (12.8)	13 (92.8)	1 (7.2)	
Iatrogenic injury	Yes	5 (4.6)	1 (20)	4 (80)	0.005
	No	105 (95.4)	87 (83)	18 (17)	
Surgery size	Medium	10 (9.1)	10 (100)	0 (00.0)	0.003
	Major	60 (54.5)	41 (68.3)	19 (31.7)	
	Supermajor	40 (36.4)	37 (92.5)	3 (7.5)	
Diagnosis	Gastric	35 (31.8)	30 (85.7)	5 (14.3)	0.019
	Biliary pancreatic	17 (15.4)	15 (88.2)	2 (11.8)	
	Small bowel	19 (17.2)	10 (52.3)	9 (47.3)	
	Colorectal	18 (16.5)	14 (77.8)	4 (22.2)	
	Appendix	21 (19.1)	19 (90.4)	2 (9.6)	
Previous laparotomy	Yes	14 (4)	7 (50)	7 (50)	0.003
	No	96 (96)	81 (84.3)	15 (15.7)	
Reoperation	Yes	7 (6.4)	0 (00)	7 (100)	0.000
	No	103 (93.6)	88 (85.4)	15 (14.6)	

The total mortality rate was 3.64%, and mortality was seen exclusively in the major abdominal surgery procedure: two patients with small bowel resection, one patient with gastrojejunostomy, and one with a colostomy.

## Discussion

Surgical complications are much more common in abdominal surgery procedures than those performed outside the abdomen, such as mastectomy and parathyroidectomy [12]. Despite the ongoing efforts to improve preoperative patient preparation and surgical techniques, putting patients at the risk of POCs is continuing, which sometimes necessitates emergency surgical intervention and high costs [13]. Additionally, identifying the risk factors may provide an opportunity to improve perioperative care by managing preoperative risk factors and lowering the risk of POCs and mortality [14].

Several studies have investigated the relationship between potential risk factors and outcomes in colorectal surgical procedures. However, only a few of these studies have examined a variety of major complications about a wide range of potential confounding factors and characterized the relative influence of these factors on specific outcomes. To address this problem, we thoroughly investigated the preoperative and operative risk factors in gastrointestinal procedures with POCs. Our goal is to determine the relationship between preoperative and operative risk factors and specific POCs. Similar studies were conducted by Manilich et al. and Sørensen et al. [9,11].

In Manilich et al.'s study, the authors just focused on the most meaningful complications of colorectal procedures, including readmission, reoperation, sepsis, anastomotic leak, small bowel obstruction, SSI, abscess, need for transfusion, and venous thromboembolism [9]. Our study was conducted in an unselected population of patients required to undergo different open gastrointestinal surgical procedures; the mortality rate within 30 days after the surgical intervention was 3.6%, and POCs occurred in 22 (20%) patients. Kim et al. reported a similar result that** **the POCs occurred in 54 patients (26%) within 30 days after surgery, and the 30-day mortality occurred in five patients (2%) [13].

We revealed a significant relationship between POCs and poor nutritional status, high ASA grade, need for blood transfusion, major abdominal operations, iatrogenic injury, reoperation, small bowel resection, and history of the previous laparotomy. In Kim et al.'s study, independent risk factors affecting 30-day POCs were older age, high ASA score, major or invasive surgeries, lower hemoglobin level, and poor nutritional status [13].

In another study, Manilich et al. investigated the factors associated with postoperative complications in patients undergoing colorectal surgery. In this study, factors were classified into two groups: those of high importance (operative time, body mass index [BMI], age, identity of the surgeon, and type of surgery) and those of low importance (sex, comorbidity, laparoscopy, and emergency). The ASA score and diagnosis were both of moderate importance. Readmission, transfusion, surgical site infection, and abscesses were the outcomes that were most influenced by variations in the critical factors. The authors concluded that the three most important factors influencing readmission rates, transfusion rates, and SSI are body mass index, operative time, and the surgeon experiences [9].

It is difficult to conclude the predictive value of POC measures by comparing the results of our study with the previous studies due to the diversity of surgical procedures, postoperative endpoints, and the variety of measures evaluated [15]. But some factors in our results were similar to those previously reported in the paper, such as high ASA grade, poor nutritional status, need for blood transfusion, major abdominal operations, reoperation, and history of the previous laparotomy [13,14]. Some factors in our study, such as smoking, male gender, emergency surgery, prolonged time of surgery, khat chewing, comorbidities (diabetes mellitus, anemia, jaundice, heart disease), and drain insertion, did not have statistically significant effects on the rate of POCs.

Sørensen et al. mentioned that some of those factors significantly affected the rate of the POCs [16]. The authors compared the predictive risk factors for POCs in open gastrointestinal surgeries within 30 postoperative days in 4,855 unselected patients with elective or emergency gastrointestinal surgeries. They reported that smoking, comorbidity, and perioperative blood loss were linked to complications after elective surgery. While male gender, peritonitis, and multiple operations were predictors of complications following emergency operations, surgical procedures have an effect on both types [16]. Another study of 164,297 colorectal resection patients discovered that chronic steroid use, overweight, severe chronic obstructive pulmonary disease (COPD), prolonged operation, emergency operations, and low serum albumin levels are strongly linked with postoperative wound disruption [17].

There are some explanations for why these factors are not significant in our study. For example, age and comorbidities are not substantial due to our study's deficient number of older patients and comorbidities. Smoking is not essential because most of our patients were recommended smoking cessation for four weeks before surgery. Additionally, 20% of our patients just operated for medium surgery such as appendectomy, and those preoperative comorbidities did not acutely affect the POC rates. This might be the reason that these factors do not significantly increase the POCs in our study, and our result should be interpreted with caution. Furthermore, our study showed that POCs after emergency surgery were more than elective procedures (14 vs 8), but statistical analysis showed no significance.

Regarding gender, the incidence of POCs, specifically anastomotic leak, is increased in males undergoing colorectal surgery, which may reflect that technical difficulty can be intensified in male patients due to their narrow pelvises [18]. Most studies revealed male predominance [11,16,19]. In our study, Kim et al. and Abebe et al.'s results demonstrated similar rates in both sexes [13,20]. This may reflect differences in study subjects and disease incidence. Additionally, the explanations for these differences are still unknown, but they may provide new insights into the mechanisms involved. Future studies with a large number are recommended to elucidate this issue.

Khat chewing is still a vague issue concerning POCs. This study did not show a significant relation between khat chewing and early POCs. There were no published studies regarding POCs and khat chewing. In another study, khat chewing was associated with intestinal obstruction, constipation, and gastritis [21]. Future studies with a large number are recommended to elucidate this issue.

Our study reveals that more POCs are observed in major surgery compared to medium-sized and supermajor-sized operations. Additionally, we showed that surgeries on small bowel were highly associated with POCs. Our result agrees with Veličković et al., which reported that POCs are positively related to major abdominal procedures [22]. Many factors influence the POCs of patients undergoing major abdominal surgery, such as malignancy, radical versus palliative surgery, intraoperative complications, and patients' comorbidities [23].

In this study, preoperative nutritional status was significantly associated with POCs, and malnourished patients had more POCs than normal patients. Our result was in agreement with the studies of Singh et al. and Fentahun et al. [24,25]. This finding could be clarified by the patients' good nutritional status, which increases growth hormone and collagen production, increases tensile strength, and lowers the risk factor of wound-healing dalliance [25]. There are no nutrition-screening services before undergoing abdominal surgery in Yemen health facilities, which renders the outcome of abdominal surgery much worse and leads to more POCs.

Our study reveals that having surgery for more than two hours does not increase the risk of developing early POCs. Our result is in contrast to that of Bayissa et al.'s study [26]. The authors demonstrated that the longer the operative time, the greater the risk of overall complications such as reoperation, medical and surgical complications, and superficial surgical site infections [26]. These discrepancies may be related to the teaching hospital and most surgeries performed by residents with attending supervision, leading to more time for performing surgical procedures.

Only a little information is available about iatrogenic injuries in colorectal surgery. The surgeon's main concerns are vessel injury, spleen damage during colorectal surgery (incidence: 0.006%), intestinal perforation, and ureteric injuries (incidence: 0.01%). Iatrogenic bowel perforation occurs either during adhesiolysis or inadvertently due to a thermic lesion, which is frequently not recognized during the operation. The surgeon should prefer primary repair or resection with anastomosis [27]. In our study, there was a significant relationship between POCs and iatrogenic injuries. A similar result was reported by Bayissa et al. [26].

In our study, drain insertion was performed in 96 (87.2%) patients and was not statistically associated with early POCs. According to recent data from a meta-analysis of randomized controlled trials, routine drainage does not confer any significant advantage in preventing postoperative complications after rectal surgery; however, it may be associated with a higher risk of postoperative bowel obstruction [28]. Additionally, adequate evidence shows that regular drainage after colorectal anastomoses does not prevent POCs [27].

In this study, the multiple postoperative complications were more than a single complication (15.5% vs 4.5%). Indeed, POCs were closely correlated and often contributed to the development of another intestinal complication. Most study results only look at the first surgical complication or compare the presence of any complication to no complication. As described by Morris et al., multiple POCs place patients at significantly increased risk of mortality and prolonged hospital stay [29]. We believe that more data on the outcomes of various complications may reveal a significant source of POCs and objectives for surgical quality improvement programs.

This study had several limitations. First, it is a retrospective single-center study. Second, only a small number of patients were studied. Third, the Clavien-Dindo grade is not used to classify POCs. Fourth, It is difficult to conclude the predictive value of POC measures by comparing the results of the previous studies due to the diversity of surgical procedures, postoperative endpoints, and the variety of measures evaluated [15]. Finally, mortality after the first 30 days postoperatively was not recorded. As a result, the mortality rate likely underestimates the actual mortality rate. Therefore, we recommend a prospective randomized study with a large number of patients to elucidate our outcome. Despite its limitations, this study provides important preliminary information on the prevalence rate and factors influencing POCs in adult patients undergoing gastrointestinal surgery in Sanaa, Yemen.

## Conclusions

There is a significant relationship between POCs and poor nutritional status, high ASA grade, need for blood transfusion, major abdominal surgeries, reoperation, small bowel resection, iatrogenic injury, and history of the previous laparotomy. These factors should be assessed when auditing surgical outcomes.
